# On Measuring the Root-Mean-Square Value of a Finite Record Length Periodic Waveform

**DOI:** 10.6028/jres.094.037

**Published:** 1989

**Authors:** E. Clayton Teague

**Affiliations:** National Institute of Standards and Technology, Gaithersburg, MD 20899

**Keywords:** periodic surface profile, periodic waveform, random error, root-mean-square value, surface roughness, uncertainty

## Abstract

An analysis of the uncertainty in measuring the root-mean-square, rms or *R*_q_, value of a periodic waveform which results from the use of a finite record length is presented. Even though the results of the analysis are somewhat as expected, i.e., that the uncertainty is inversely proportional to the number of periods in the record, the explicit relationship between the magnitude of the uncertainty and properties of the waveform does not appear to be available in the literature. The paper first presents an introductory example in terms of the reasonably well known case of bandwidth limited Gaussian waveform to introduce definitions. Following this is an analysis of the periodic waveform using the same approach. It is shown that for a large number of periods, *n*, in the record length, the normalized three standard deviation of the rms value is given by 3/(8*πn*).

## 1. Introduction

This paper presents a discussion of the uncertainty in measuring the root-mean-square, rms, value of a periodic waveform which results from the use of a finite record length. The analysis was motivated by seeking to understand the source of a random uncertainty component which was present in some measurements of the absolute arithmetic average, *R*_a_, deviation from a mean line of profiles of precision roughness specimens. The profiles of these specimens had an approximately triangular waveform with two wavelengths and amplitudes. For the longer wavelength specimens the random phasing of the waveform with respect to the recording interval proved to be a major source of uncertainty in the measurements. In this case the record length included 40 periods of the waveform. Even though the results of the analysis are somewhat as expected, i.e., that the uncertainty is inversely proportional to the number of periods in the record, the explicit relationship between the magnitude of the uncertainty and properties of the waveform does not appear to be available in the literature. The discussion first presents an example in terms of the reasonably well known case of a bandwidth limited Gaussian waveform to introduce some definitions and relationships. Following this is an analysis of the periodic waveform which uses a similar approach.

## 2. Bandwidth Limited Gaussian Waveform

By definition the mean square value, *q*^2^, calculated from a finite length *L* of the waveform *y*(*x*) is given by the equation:

$$q^{2}=1/L\int_{0}^{L}y^{2}\left(x\right)dx.$$ (1)

For convenience and brevity in the following discussion, the definitions listed below will be used [1].

Sample

space≡the set of points representing the possible outcomes of a measurement.
ϕ(*k*)≡a real number, called the random variable, which represents the outcome of a measurement indexed by *k*.
*p*(ϕ)≡lim Probability $\frac{\left[\phi <\phi \left(k\right)<\phi +\Delta \phi \right]}{\Delta \phi }$ ∆ϕ→0
*E*{g[ϕ(*k*)]}≡the expected value of any real single-valued continuous function g(ϕ) of the random variable ϕ(*k*). It is given by:
  $$E\left\{g\left[\phi \left(k\right)\right]\right\}=\int_{-\infty }^{+\infty }g\left(\phi \right)p\left(\phi \right)d\phi .$$

As an example, the mean square value of *y*(*x*) is given by:

$$E\left[y^{2}\left(x\right)\right]=\int_{-\infty }^{+\infty }y^{2}\left(x\right)p\left(x\right)dx.$$

*E*[*y*^2^(*x*)] will be defined as ${\bar{q}}^{2}$ which is that value approached by *q*^2^ as the record length *L* approaches infinity. Also ${\bar{q}}^{2}$ is the limiting value for the mean *q*^2^ as the number of samplings gets large. The variance of *y*(*x*) is defined by:

$$\text{var}\left\{y\right\}=E\left[{\left(y-\bar{y}\right)}^{2}\right]=\int_{-\infty }^{+\infty }{\left(y-\bar{y}\right)}^{2}p\left(x\right)dx.$$

Similarly the variance in *q*^2^ is defined by:

$$\text{var}\left\{q^{2}\right\}=E\left[{\left(q^{2}-{\bar{q}}^{2}\right)}^{2}\right].$$

For the special case when *y*(*x*) is a bandwidth limited Gaussian waveform with zero mean, Bendat and Piersol [1] show that

$$\begin{array}{l} \frac{\text{var}\left\{q^{2}\right\}}{{\bar{q}}^{4}}=\frac{2L*}{L}\left[1-e^{-2L/L*}\right]\\ \;+\frac{L*}{L}\left[\left(2\frac{L}{L*}+1\right)e^{-2L/L*}-1\right], \end{array}$$

where *L* * is the shift distance required for the autocorrelation function to drop by 63% of its zero shift value. If *L* > >L* the relation reduces to

$$\frac{\text{var}\left\{q^{2}\right\}}{{\bar{q}}^{4}}=2\frac{L*}{L}.$$

The propagation of error formulae discussed by Ku [2] may be used to relate the variance of the root-mean-square $\text{value}=\text{rms}=\sqrt{q^{2}}$ to this variance of *q*^2^. Results given in table 1 of this reference show that if

$$\frac{\text{var}q^{2}}{{\bar{q}}^{4}}=\epsilon ,\text{then}\frac{\text{var}\;\text{rms}}{{\bar{q}}^{2}}=\frac{\epsilon }{4}.$$

Thus, the normalized three-standard-deviations limit, 3SD, in the root-mean-square values calculated from randomly sampled lengths, *L*, of a Gaussian profile with a correlation length L* is given by:

$$3\text{SD}\;\text{limit}\;\text{of}\frac{\text{rms}}{\bar{q}}=\frac{3}{2}\frac{\sqrt{2L*}}{L}=2.1\frac{\sqrt{L*}}{L}.$$

Whitehouse and Archard [3] report reasonable confirmation of this result for their test specimens which had approximately Gaussian profiles. The importance of this result is stated very clearly by Whitehouse and Archard [3]. "The variance of measured rms or R_a_ values for the roughness of a surface may be found easily if one knows the standard deviation of a large number of such measurements made upon the same surface. Alternatively one may predict the variance from a knowledge of the correlation length of a typical profile of the test surface." The argument just given for the 3SD limit partially fulfills the "it can be shown" statement by Whitehouse and Archard.

## 3. Periodic Waveforms

Unfortunately these results do not apply for periodic waveforms such as that of many precision roughness specimens. However, the principles of calculation are still applicable. Consider the example of the sinusoidal waveform:

y(x)=Asin(ωx+θ). (2)

The waveform is sampled for a length *L* with the phase angle *θ* considered as the random variable of the sample space. The angle *θ* will be considered as having a uniform distribution over values from 0 to 2*π*, i.e.,

$$p\left(\theta \right)=\frac{1}{2\pi },0<\theta <2\pi \;\text{and}\;p(\theta )=0,\text{for all other values of}\;\theta$$

According to definitions in eqs (1) and (2):

$$q^{2}=\frac{1}{L}\int_{0}^{L}A^{2}\sin^{2}\left(\omega x+\theta \right)dx.$$

Evaluation of the integral yields

$$q^{2}=\frac{A^{2}}{2}\left[1-\frac{\sin\;2\left(\delta +\theta \right)}{2\omega L}+\frac{\sin\;2\theta }{2\omega L}\right],$$

where *δ = ωL−* 2*nπ*. The conditional variance of *q*^2^, given *δ*, is (noting that ${\bar{q}}^{2}=A^{2}/2$):

$$\begin{array}{l} \mathrm{var}\left(q^{2}|\delta \right)=E\left[{\left(q^{2}-{\bar{q}}^{2}\right)}^{2}\right]=-\frac{1}{2\pi }\\ \times \int_{0}^{2\pi }{\left[\frac{A^{2}}{2}(1-\frac{\sin\;2\left(\delta +\theta \right)}{2\omega L}+\frac{\sin\;2\theta }{2\omega L}-\frac{A}{2}\right]}^{2}d\theta . \end{array}$$

Evaluation of the integral yields:

$$\mathrm{var}\left(q^{2}|\delta \right)=\frac{A^{4}\left(1-\cos\;2\delta \right)}{16\omega^{2}L^{2}}.$$

Thus, if the record length happens to include an integral number of periods, var *q*^2^ *= 0*. But if we take the more likely case in which 8 has a distribution of values like that of *θ* due either to variations in the measured wavelength, ω^−1^, or in the record length, *L*, the result integrated with respect to 8 is obtained as follows.

$$\begin{array}{l} \mathrm{var}{\left(q^{2}\right)}_{\delta }=\frac{1}{2\pi }\int_{0}^{2\pi }\mathrm{var}\left(q^{2}|\delta \right)d\delta \\ =\frac{1}{2\pi }\int_{0}^{2\pi }\frac{A^{4}\left(1-\cos2\delta \right)}{16\omega^{2}L^{2}}d\delta . \end{array}$$

Substituting *ωL = δ +* 2*nπ* followed by the introduction of dummy variables *x* and *y* reduces the integral to:

$$\begin{array}{l} \mathrm{var}{\left(q^{2}\right)}_{\delta }=\frac{1}{2\pi }\int_{2n\pi }^{2\left(n+1\right)\pi }x^{-2}dx\\ -\frac{1}{\pi }\int_{4n\pi }^{4\left(n+1\right)\pi }\left(\cos\;y/y^{2}\right)dy. \end{array}$$

Solving these integrals yields:

$$\begin{array}{l} {\left(\mathrm{var}\;q^{2}\right)}_{\delta }=\frac{A^{4}}{16}\frac{1}{4\pi^{2}n\left(n+1\right)}\text{or}\frac{{\left(\mathrm{var}\;q^{2}\right)}_{\delta }}{{\bar{q}}^{4}}\\ =\frac{1}{16\pi^{2}n\left(n+1\right)} \end{array}$$

where *n* is again defined by the equation *δ = ωL−*2*nπ*. This result for (var *q*^2^)δ does account for the relationship between *ωL* and δ Use of the same relationships between var *q*^2^ and var rms given earlier yields:

$$\frac{{\left(\text{var}\;\text{rms}\right)}_{\delta }}{{\bar{q}}^{2}}=\frac{1}{64\pi^{2}n\left(n+1\right)}.$$

An *averaged* normalized three-standard-deviation limit for variations of the rms values is then given by:

$$3\text{SD}\left(\frac{\text{rms}}{\bar{q}}\right)=\frac{3}{8\pi \sqrt{n\left(n+1\right)}},$$ (3)

or for large *n*,

$$3\text{SD}\left(\frac{\text{rms}}{\bar{q}}\right)=\frac{3}{8\pi n}.$$ (4)

Finally, if the record length *L* and measured wavelengths, *ω*^−1^, are stable and such that δ has a narrow distribution about *π*/2 or 3π/2 the $3\text{SD}\left(\text{rms}/\bar{q}\right)$ could be as much as twice this average value.

## 4. Comparison with Variations of Roughness Measurements

For surface profiles obtained from roughness measurements, one would expect that the normalized 3SD of a set of measured *R*_a_ values would be approximately equal to that computed for the *R*_q_ (rms) values since both quantities are similar measures of the sampled profile's empirical probability density function. The measurements of *R*_a_ values which initiated this analysis should therefore be understandable in terms of the results just derived.

For the measurements, two types of specimens were used; one set had an *R*_a_ value of 3.2 *µ*m and a wavelength such that *n* = 40; the other set had an *R*_a_ value of 0.5 *µ*m and a wavelength such that *n* = 250. Record lengths for the measurements were approximately 3.8 mm. Predicted uncertainties for the variations of a measured value about the mean *R*_a_ are therefore:

$$3{\text{SD}}_{3.2\mu m}=0.3\%\;\text{of}\;\text{mean}\;R_{a},$$

and

$$3{\text{SD}}_{0.5\mu m}=0.05\%\;\text{of}\;\text{mean}\;R_{a},$$

if one assumes the *averaged* three standard deviations limit. The *maximum* values for the analytical uncertainties are 0.6 and 0.1% of the mean *R*_a_ values for the 3.2 and 0.5 *µ*m specimens, respectively.

In a series of 80 sets of three *R*_a_ measurements, the uncertainty (3 standard deviations) of these triplicate measurements with respect to their average values was:

$$3{\text{SD}}_{3.2}=1.0\%\;\text{of}\;\text{mean}\;R_{a},$$

and

$$3{\text{SD}}_{0.5}=0.7\%\;\text{of}\;\text{mean}\;R_{a}.$$

The intent of this comparison between the analytical and experimental values is not that of justifying the theoretical analysis. However, in terms of understanding the experimental variations the agreement is sufficient to confirm that, for the longer wavelength specimen, a major source of uncertainty for the roughness measurements results from the finite sampling length.

**Figure 1:**
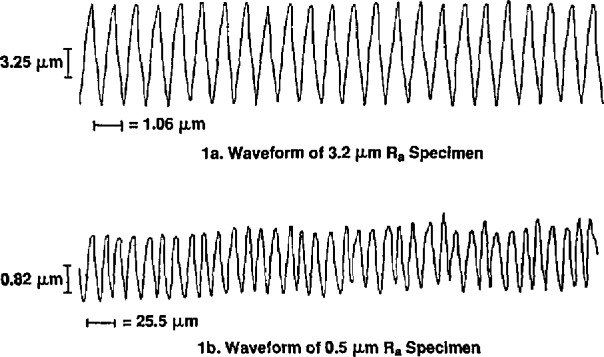
Waveforms of roughness specimens which were used for statistical studies.

The imperfect waveforms of the precision roughness specimens are the most likely sources of the residual uncertainty. As illustrated in figure 1 the specimen waveforms have distortions which are produced by either an additive random function together with phase modulation or by the combination of random amplitude and phase modulation. The effect of an additive random function is the only one which can be readily analyzed. For this case the rms value of the sum of two uncorrelated waveforms adds in a rms manner. Assume that the waveforms can be represented by the sum of a random component, with an rms value of *q*_R_ and a correlation length of L*, and a periodic component, with an rms value of *q*_p_ and angular frequency ω. Then the use of propagation of error formulae derived by Ku [2] yields:

$$3\text{SD}\left(\frac{\text{rms}}{\bar{q}}\right)=1.5\left[2a\frac{L*}{L}+\frac{b}{16n^{2}\pi^{2}}\right]$$

where *n* and *L* are as defined earlier and

$$a={\left(1+\frac{q_{R}^{2}}{q_{P}^{2}}\right)}^{-2},\;b={\left(1+\frac{q_{P}^{2}}{q_{R}^{2}}\right)}^{-2}.$$

Estimates of $q_{R}^{2}$ and *L* * from the calculation of autocorrelation functions of the waveforms for the 0.5*µ*m—*R*_a_ specimen are: L* = 0.05 *L* and $q_{R}=0.01\;q_{P}^{2}$. Effects of random amplitude and phase modulation are not accounted for in these estimates. With these values of $q_{R}^{2}$ and *L* * the dominant source of uncertainty for the waveform sampled for 250 periods is the random additive component which increases the uncertainty limits to 0.3% of the mean value. Approximately the same values for L* and $q_{R}^{2}$ were obtained from studies of the 3.17*µ*m —*R*_a_ specimen. Since $q_{R}^{2}=2.8\times 10^{-4}q_{P}^{2}$, in this instance, the uncertainty produced by the random component is insignificant for measurements on this specimen.

## 5. Conclusion

The uncertainty in measuring the root-mean-square value of a finite record length of a periodic waveform has been obtained and is given in eqs (3) and (4). The theoretical results were compared with data obtained from surface profile measurements of precision roughness specimens. However, eqs (3) and (4) are quite general and will apply to rms measurements of the amplitude of periodic waveforms arising from other areas.

During customary announcements of this paper to NIST staff, an analysis of the errors of measurement due to variations in sampling interval length, (complimentary to that presented here), was brought to the author's attention. The effects of adjusting the sample interval length relative to an integral number of cycles of the test waveform are determined and applied to measurements of electrical power with a wattmeter [4].
